# First report of a chrysovirus infecting a member of the fungal genus *Ilyonectria*

**DOI:** 10.1007/s00705-022-05551-2

**Published:** 2022-08-13

**Authors:** Tom P. Pielhop, Carolin Popp, Dennis Knierim, Paolo Margaria, Edgar Maiß

**Affiliations:** 1 Institute of Horticultural Production Systems, Department of Phytomedicine, Leibniz University Hannover, Herrenhäuser Str. 2, 30419 Hannover, Germany; 2 Leibniz Institute DSMZ, German Collection of Microorganisms and Cell Cultures, Inhoffenstraße 7B, 38124 Brunswick, Germany

## Abstract

The fungus *Ilyonectria pseudodestructans* belongs to the family *Nectriaceae* and was found to be part of the endophytic microbiome of apple trees (*Malus* x *domestica*, Borkh.) with apple replant disease (ARD). After dsRNA extraction, a mycoviral infection became evident. Here, we report the identification of a new virus, tentatively named “Ilyonectria pseudodestructans chrysovirus 1” (IpCV1), as the first member of the proposed new species “*Alphachrysovirus ilyonectriae*” within the genus *Alphachrysovirus*. This is the first report of a chrysovirus infecting a member of the fungal genus *Ilyonectria.* IpCV1 has a tripartite dsRNA genome with a total length of 8944 bp. The segments are 3439 bp, 2850 bp, and 2655 bp in length, and each dsRNA carries a single ORF. The encoded viral proteins are a 125.92-kDa RNA-dependent RNA polymerase, a 100.75-kDa coat protein, and one protein of unknown function with a predicted molecular mass of 93.04 kDa. The 5´ and 3´ UTRs are comparatively short and are 79 to 91 bp and 62 to 148 bp in length, respectively. This study provides the basis for further investigations of the impact of IpCV1 on its host and the etiology of ARD.

**Supplementary Information:**

The online version contains supplementary material available at 10.1007/s00705-022-05551-2.

Apple replant disease (ARD) is a worldwide problem that occurs in orchards after apple trees are replanted repeatedly on the same site. ARD is caused by a changed (micro-) biome and the subsequent reaction by the plant [1]. The complex combination of factors causing ARD is not yet well understood. The virus, tentatively named "Ilyonectria pseudodestructans chrysovirus 1" (IpCV1), which, in the course of this work, was identified as a member of a new species for which we propose the name *Alphachrysovirus ilyonectriae*, is part of this complex as a mycovirus infecting the fungus *Ilyonectria pseudodestructans*. For proposing the names of the virus and its species, the new ICTV guideline on binomial virus species nomenclature was taken into account [2].

The fungus itself was isolated from M26 apple roots (*Malus* x *domestica*, Borkh., *in vitro* propagated), which were part of a central experiment described by Mahnkopp et al. [3]. The plants were grown for eight weeks in soil from a site in Heidgraben (53.699199, 9.683171; Schleswig-Holstein, Germany) where ARD was observed. Before fungal isolation, the apple root surfaces were disinfected and 1-cm root pieces were plated in water agar. The isolation and identification of the fungus were carried out as described by Popp et al. [4]. The isolate of *I.* *pseudodestructans* used in this study was designated as N7. After nucleic acid extraction, N7 was identified by PCR and sequencing of multiple loci of the fungal genome, since sequencing of the internal transcribed spacer (ITS) alone is not sufficient for distinguishing *Nectriaceae* species [5]. The loci were chosen according to Cabral et al. [6]. In addition to the ITS, the HIS (histone H3 gene), TEF (translation elongation factor 1-alpha), and TUB (β-tubulin gene) loci were sequenced.

To analyze the fungal virome, dsRNA was extracted according to a modification of the protocol of Morris and Doods [7] as described by Lesker et al. [8], and 20 mL of the eluate was digested with 20 U of RNase T1 (Roche) and 40 U of DNase I (Roche). Subsequent purification and concentration led to a final elution volume of 3 mL. For further analysis, dsRNA was precipitated with ethanol from 500 µL of eluate and dissolved in 20 µL of water. The purified dsRNA was subjected to electrophoresis in a 1% agarose gel, and three fragments were separated. A photograph of the gel is available in ESM1. Following random cDNA synthesis and second-strand synthesis with random octamer primers, an Illumina Nextera XT library was prepared and sequenced on a NextSeq device as paired-end reads (2 × 151 bp) at the Leibniz Institute DSMZ. Trimming of raw reads (188,324) and *de novo* assembly were performed using the bioinformatics tools from the BBtools package (https://sourceforge.net/projects/bbmap/) implemented in Geneious R11.1 and 2022.1.1 (Biomatters, Auckland, New Zealand). The assembled contigs were aligned against a custom NCBI nuclear-core reference database using BLASTn and BLASTp algorithms, and three of the contigs could be assigned to a tripartite chrysovirus. An overview of the number of reads mapping the genomic RNAs of IpCV1 is given in the online resource ESM2.

The terminal dsRNA ends were determined using an adapted RACE protocol by the method of Frohman et al. [9]. Both 3’ ends of the dsRNA (sense and antisense) were determined for all three viral segments. After synthesis, 3 µL of cDNA was tailed using 20 U of terminal deoxynucleotidyl transferase (TdT; Thermo Fisher Scientific, Waltham, MA, USA) and a final concentration of 5 mM of dATP, dCTP, dGTP, or dTTP (Thermo Fisher Scientific, Waltham, MA, USA) in at least two different reactions for each of the RNA ends.

The RACE reactions resulted in the completion of the full genomic sequence of IpCV1, with a total size of 8944 bp, separated into three segments with one ORF each. Segment 1 (S1) has a length of 3439 bp and an ORF of 3205 bp, encoding a 125.92-kDa RNA-dependent RNA polymerase. Segment 2 (S2) is 2850 bp in length, with a 2696-bp ORF, encoding a coat protein with a mass of 100.75 kDa. Segment 3 (S3) has a length of 2655 bp and a 2495-bp ORF. The function of the 93.04-kDa protein encoded by ORF 3 is unknown. The 5’ UTRs consist of 85 bp (S1), 91 bp (S2), and 79 bp (S3), whereas the lengths of the 3’ UTRs are 148 bp (S1), 62 bp (S2), and 80 bp (S3). A scaled genome map of IpCV1 is shown in Fig. 1a.

**Fig. 1:**
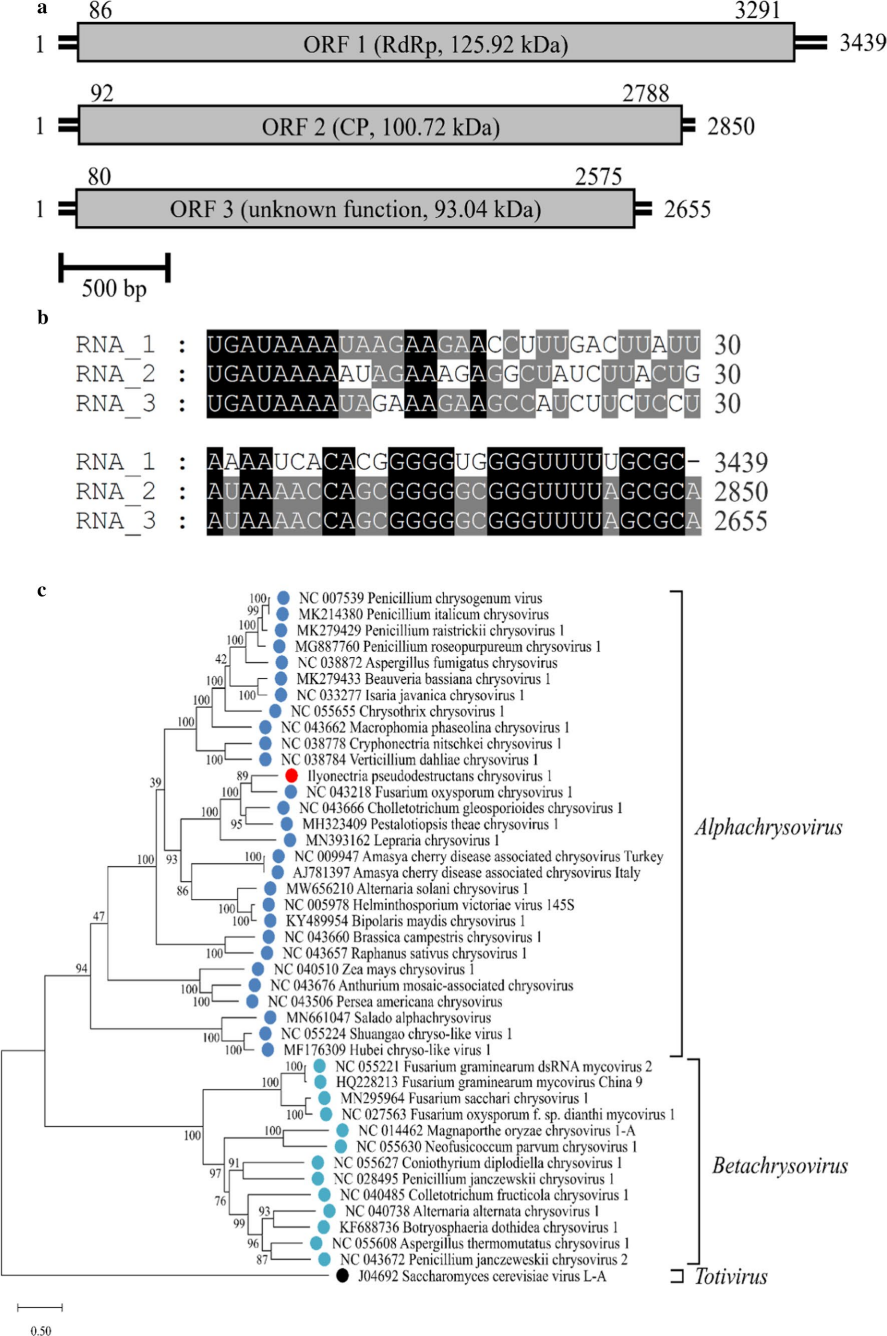
**a** Scaled genome map of IpCV1. Numbers represent nucleotide positions of the 5’ ends, ORFs, and 3’ ends. Boxes represent ORFs, with the encoded protein indicated. **b** Alignment of 5’- and 3’-UTRs. Numbers next to the sequences represent nucleotide positions. Mapped with GeneDoc 2.7. **c** Maximum-likelihood tree based on RdRp aa sequences of chrysoviruses and a totivirus, constructed in MEGA X with 1000 bootstrap replicates, the Le_Gascuel_2008 substitution model with gamma-distributed rates, invariant sites, and empirical base frequencies (LG+G+I+F). For the alignment, the MUSCLE algorithm was used with default parameters (gap opening, -2.9; gap extension, 0) [8, 10, 11]

Alignments of the terminal sequence regions showed conserved sequences at both UTRs. The first eight nucleotides at the 5’ end (5’-UGAUAAAA) are identical in all segments. Moreover, the 5’ UTRs of most chrysoviruses share another conserved region (Box1), followed by CAA repeats [10]. IpCV1 has comparatively short UTRs, and the Box1 and CAA_(n)_ motifs are present but rather short as well. The 3’ ends of S2 and S3 share 13 conserved nucleotides (GGGUUUUWGCGCA-3’), whereas S1 shares only 12 of them, lacking the terminal A. The alignments were visualized using the software GeneDoc 2.7 (National Resource for Biomedical Supercomputing, 300 South Craig Street, Pittsburgh, PA, USA) and are shown in Fig. 1b.

Phylogenetic analysis was performed using sequences from a set of 28 alphachrysoviruses and 13 betachrysoviruses together with a totivirus as an outgroup. A sequence alignment of the amino acid (aa) sequences of the RdRp was performed using MUSCLE, and a phylogenetic tree was constructed as described by Kotta-Loizou et al., using the software MEGA X [10–12]. The bootstrap method was applied with 1000 replications, and the Le_Gascuel_2008 substitution model with gamma rates, invariant sites, and empirical base frequencies (LG+G+I+F) was used [13]. In the phylogenetic tree (Fig. 1C), Fusarium oxysporum chrysovirus 1 (FoCV1) appeared to be the closest relative of IpCV1, and both were clustered together with Colletotrichum gloeosporioides chrysovirus 1 (CgCV1), Pestalotiopsis theae chrysovirus 1 (PtCV1), and Lepraria chrysovirus 1 (LiCV1) of the genus *Alphachrysovirus*, with 100% bootstrap support. This was supported by the results of pairwise identity analysis using the EMBOSS/Needle tool [14]. In a comparison of the aa sequences of RdRp and CP of IpCV1 and the alphachrysoviruses used for phylogenetic analysis, IpCV1 has the highest sequence identity to CgCV1 (RdRp: 60.9%, CP: 39.3%) and PtCV1 (RdRp: 56.2%, CP: 38.9%). However, the aa sequence of the RdRp of FoCV1 shows less sequence identity to IpCV1 (50.5%) despite the closeness of these viruses in the phylogenetic tree. These seemingly conflicting results can be explained by the relatively short RdRp sequence of FoCV1, which led to a large number of gaps in the pairwise alignment (24%) when calculating the identity score. Furthermore, only a partial CP sequence is available for FoCV1, and therefore, no complete comparison could be made. An overview of the highest identity scores is given in Table 1. An overview of all calculated sequence identity values can be found in the online resource ESM3. According to the ICTV species demarcation criteria, the identity has to be ≤ 70% and ≤ 53% for the aa sequences of RdRp and CP [11]. Therefore, IpCV1 is suggested to be a member of a proposed new species in the genus *Alphachrysovirus*, family *Chrysoviridae*, for which we propose the name "*Alphachrysovirus ilyonectriae*", according to the new ICTV guidelines on binomial virus species nomenclature [2].

**Table 1:** Sequence identity (%) of IpCV1 with the six highest-scoring chrysoviruses, calculated using the EMBOSS/Needle software [12]

Virus	Accession no.	Identity RdRP (%)		Accession no.	Identity CP (%)	
		ORF (nt)	aa		ORF (nt)	aa
IpCV1	OM993531	–	–	OM993532	–	–
CgCV1	NC_043666	62.4	60.9	NC_043668	52.5	39.3
PtCV1	MH323409	59.4	56.2	MH323410	51	38.9
HvV145S-A9	NC_005978	51.4	40.9	NC_005979	46.6	25.5
BmCV1	KY489954	50.9	41	KY489955	48.1	25.8
CCRSACV	AJ781397	50.9	36.4	AJ781398	47.5	26.7
AsCV1	MW656210	50.8	39.6	MW656211	48.1	27.3

Percent identity values for the nucleotide (ORF) and deduced aa sequences of the RdRp and CP are shown

Since the identification of Penicillium chrysogenum virus as the first member of this genus, new chrysoviruses have been discovered continually [15, 16]. This is the first report of a chrysovirus infecting a member of the fungal genus *Ilyonectria*. Future studies are needed to determine whether there is a hypo- or hypervirulent effect of IpCV1 on its host, since it has been shown that chrysoviruses can have a hypovirulent effect [17], and the ability to induce hypervirulence has been reported for other mycoviruses [18]. The findings of these studies suggest possible disease-mitigation strategies involving the control or application of such viruses [17].

## Sequences

The full genomic sequences of Ilyonectria pseudodestructans chrysovirus 1 (IpCV1) identified in this study were uploaded to the NCBI GenBank database and are available under the following accession numbers, as soon as the study is in print:

IpCV1 segment 1: OM993531

IpCV1 segment 2: OM993532

IpCV1 segment 3: OM993533

## Supplementary Information

Below is the link to the electronic supplementary material.

Supplementary file1 (PDF 98 KB)

Supplementary file2 (PDF 13 KB)

Supplementary file3 (PDF 46 KB)

Supplementary file4 (FASTA 3 KB)

Supplementary file5 (FASTA 3 KB)

Supplementary file6 (FASTA 3 KB)
