# Supplementary material for: First report of a chrysovirus infecting a member of the fungal genus Ilyonectria
Source: Arch Virol. 2022 Aug 13;167(11):2411–5. doi: 10.1007/s00705-022-05551-2 (PMC9556398; doi:10.1007/s00705-022-05551-2)
Supplement: Supplementary file 1 — Supplementary file1 (PDF 98 KB) [file 705_2022_5551_MOESM1_ESM.pdf]

Article title:

First report of a chrysovirus infecting a member of the fungal genus *Ilyonectria*

Journal:

Archives of Virology

Authors:

Tom P. Pielhop, Carolin Popp, Dennis Knierim, Paolo Margaria, Edgar Maiß

Corresponding author:

Tom P. Pielhop, pielhop@ipp.uni-hannover.de

Institute of Horticultural Production Systems, Dept. Phytomedicine, Leibniz University Hannover, Herrenhäuser Str. 2, 30419, Hannover, Germany.

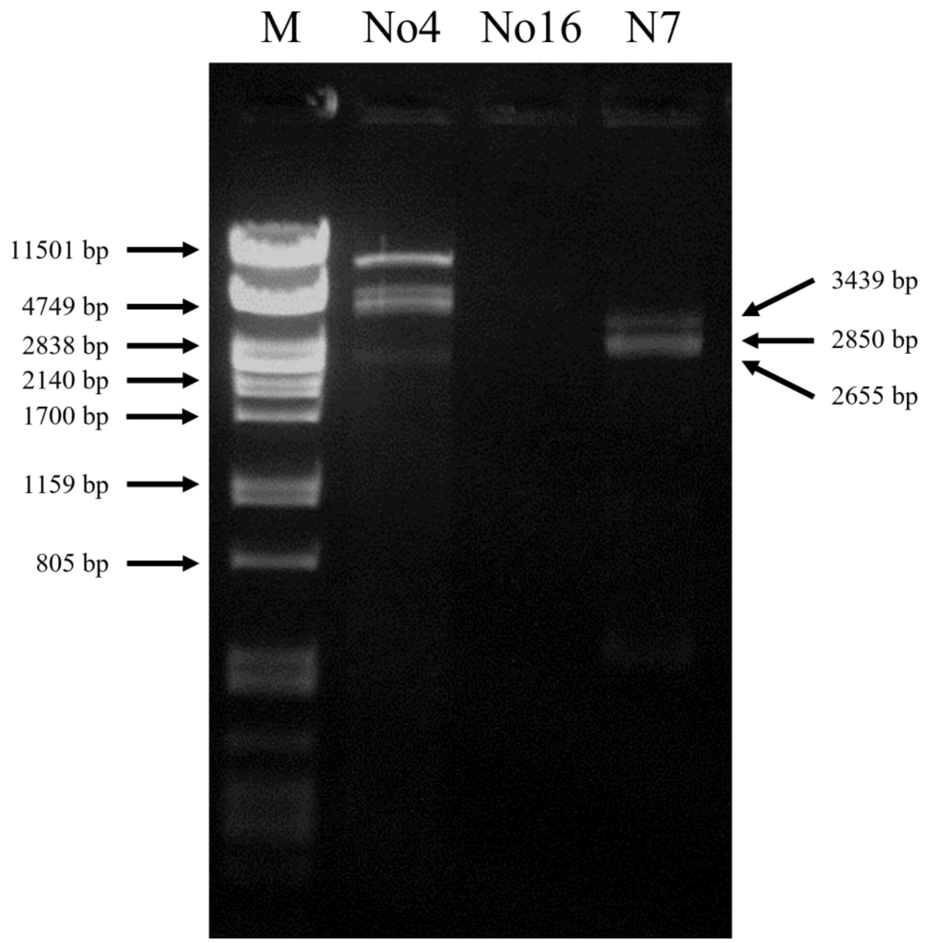

Fig. ESM1: 1 % agarose gel with dsRNA of different fungal isolates. Lines: **M**: DNA ladder (Phage lambda DNA, PstI digested); No4: *Rugonectria rugulosa;* No16: *Dactylonectria torresensis*, **N7: *Ilyonectria pseudodestructans*.**
