# Supplementary material for: First report of a chrysovirus infecting a member of the fungal genus Ilyonectria
Source: Arch Virol. 2022 Aug 13;167(11):2411–5. doi: 10.1007/s00705-022-05551-2 (PMC9556398; doi:10.1007/s00705-022-05551-2)
Supplement: Supplementary file 2 — Supplementary file2 (PDF 13 KB) [file 705_2022_5551_MOESM2_ESM.pdf]

Article title:

First report of a chrysovirus infecting a member of the fungal genus *Ilyonectria*

Journal:

Archives of Virology

Authors:

Tom P. Pielhop, Carolin Popp, Dennis Knierim, Paolo Margaria, Edgar Maiß

Corresponding author:

Tom P. Pielhop, pielhop@ipp.uni-hannover.de

Institute of Horticultural Production Systems, Dept. Phytomedicine, Leibniz University Hannover, Herrenhäuser Str. 2, 30419, Hannover, Germany.

Tab. ESM2: Number of reads mapping the genomic RNAs of IpCV1. A total of 188,324 reads were generated from the sequencing of the library.

Virus	Genomic RNA	Length (nt)	Mapped reads	% of mapped reads
IpCV1	1	3439	1508	0.80
IpCV1	2	2850	1410	0.75
IpCV1	3	2655	1687	0.90
