# Supplementary material for: First report of a chrysovirus infecting a member of the fungal genus Ilyonectria
Source: Arch Virol. 2022 Aug 13;167(11):2411–5. doi: 10.1007/s00705-022-05551-2 (PMC9556398; doi:10.1007/s00705-022-05551-2)
Supplement: Supplementary file 3 — Supplementary file3 (PDF 46 KB) [file 705_2022_5551_MOESM3_ESM.pdf]

Article title:

First report of a chrysovirus infecting a member of the fungal genus *Ilyonectria*

Journal:

Archives of Virology

Authors:

Tom P. Pielhop, Carolin Popp, Dennis Knierim, Paolo Margaria, Edgar Maiß

Corresponding author:

Tom P. Pielhop, pielhop@ipp.uni-hannover.de

Institute of Horticultural Production Systems, Dept. Phytomedicine, Leibniz University Hannover, Herrenhäuser Str. 2, 30419, Hannover, Germany.

Tab. ESM3: Sequence identity (%) of IpCV1 with the alphachrysoviruses, calculated with the EMBOSS/Needle software [12]. Sequence length and identity percentages of the nucleotide (ORF) and deduced aa sequence of the RdRp and CP are shown.

		ORF (RdRp)		aa sequence (RdRp)			ORF (CP)		aa sequence (CP)	
	Acc. no	Length (nt)	Identity (%)	Length (aa)	Identity (%)	Acc. No	Length (nt)	Identity (%)	Length (aa)	Identity (%)
**IpCV1**	OM993531	3291	-	1096	**-**	OM993532	2697	-	898	**-**
**CgCV1**	NC_043666	3267	**62.4**	1088	**60.9**	NC_043668	2703	**52.5**	900	**39.3**
**PtCV1**	MH323409	3132	**59.4**	1043	**56.2**	MH323410	2706	**51**	901	**38.9**
**HvV145S-A9**	NC_005978	3261	**51.4**	1086	**40.9**	NC_005979	2688	**46.6**	895	**25.5**
**BmCV1**	KY489954	3267	**50.9**	1088	**41**	KY489955	2685	**48.1**	894	**25.8**
**CCRSACV**	AJ781397	3264	**50.9**	1087	**36.4**	AJ781398	2982	**47.5**	993	**26.7**
**AsCV1**	MW656210	3255	**50.8**	1084	**39.6**	MW656211	2718	**48.1**	905	**27.3**
**MpCV1**	NC_043662	3318	**50.5**	1105	**36.9**	NC_043664	2976	**46.3**	991	**22.1**
**PcV**	NC_007539	3354	**50.4**	1117	**36**	NC_007540	2949	**43.6**	982	**20.5**
**ACDACV**	NC_009947	3264	**50.1**	1087	**36.3**	NC_009946	2985	**46.7**	994	**26.1**
**AfCV**	NC_038872	3345	**49.9**	1114	**36.4**	NC_038873	2862	**45.3**	953	**24**
**CCV1**	NC_055655	3270	**49.9**	1089	**35.4**	NC_055656	2868	**45.8**	955	**24.2**
**IjCV-1**	NC_033277	3354	**49.9**	1117	**35.3**	NC_033317	2925	**46.1**	974	**25**
**BbCV1**	MK279433	3348	**49.4**	1115	**35.8**	MK279434	2931	**45.4**	976	**24.1**
**PrCV1**	MK279429	3351	**49.3**	1116	**36.6**	MK279430	2952	**45.5**	983	**23.4**
**VdCV1**	NC_038784	3327	**49.3**	1108	**36.6**	NC_038782	3051	**43.3**	1016	**19.7**
**PiCV1**	MK214380	3354	**49.2**	1117	**36**	MK214381	2949	**44.3**	982	**21.7**
**PrpCV1**	MG887760	3354	**48.8**	1117	**37.1**	MG887761	2702	**44.6**	900	**21.9**
**BcCV1**	NC_043660	3441	**48.5**	1146	**33**	NC_043659	3351	**43.7**	1116	**20.2**
**RsCV1**	NC_043657	3417	**48.5**	1138	**31.6**	NC_043656	3306	**41.1**	1101	**20.4**
**AMAV**	NC_043676	3300	**47.1**	1099	**27.6**	NC_043677	2994	**44.2**	997	**16.8**
**ZmCV1**	NC_040510	3273	**47.1**	1090	**27.6**	NC_040509	3981	**40.5**	1326	**14.6**
**CnCV1**	NC_038778	2889	**46**	962	**32.7**	NC_038779	2721	**45.5**	906	**22.6**
**PaCV**	NC_043506	3282	**45.5**	1093	**27.5**	NC_043508	3141	**44.8**	1046	**17.1**
**LiCV1**	MN393162	2539	**45**	846	**38.9**	MN393161	2316	**48.1**	772	**30.4**
**SV**	MN661047	3363	**44.8**	1120	**24.6**	MN661048	2850	**42.7**	949	**14.1**
**SCLV**	NC_055224	3357	**44.4**	1118	**24.9**	NC_055227	2709	**42.5**	902	**15.9**
**HCLV**	MF176309	3357	**44.3**	1118	**23.7**	MF176310	2853	**45.1**	950	**13.8**
**FoCV1**	NC_043218	2553	**49.4**	851	**50.5**	NC_043219	648	Sequence incomplete		
**PbcV**	MG887763	1620	**29.8**	539	**9.6**	No GenBank entry		**NA**	NA	**NA**
